# Toward a Latin American Cancer Observatory

**DOI:** 10.1200/JGO.2015.000844

**Published:** 2015-11-25

**Authors:** Eduardo Cazap, Mauricio Magalhães Costa, Abelardo Meneses García, Raúl Murillo Moreno, Eva M. Ruiz de Castilla Yabare, Pablo Sitic Vargas, Ignacio Zervino

**Affiliations:** Latin-American and Caribbean Society of Medical Oncology, Buenos Aires, Argentina; Federación Latinoamericana de Mastología, Rio de Janeiro, Brazil; Instituto Nacional de Cancerología, Mexico City, México; Instituto Nacional de Cancerología, Bogota, Colombia; Esperantra, Lima, Perú; Instituto Oncológico del Oriente Boliviano, Santa Cruz de la Sierra, Bolivia; Fundación Atención Comunitaria Integral al Paciente Oncológico, Buenos Aires, Argentina

Cancer affects more than one million Latin Americans every year.^1,2^ And in tandem with the global trend, its incidence in the region is increasing, led mainly by factors such as urbanization, population aging, and the so-called westernization of lifestyles. If current trends continue, 95% of the world's population will be living in large cities in 30 years. Urbanism, a group of conditions determined by life in cities, is an important factor in cancer development. Population aging and the westernization of lifestyles as seen in China are also producing a profound epidemiologic transition that is increasing the incidence and mortality rates of cancer.

The factors mentioned above, among others, influence the Human Development Index, a composite statistic by the United Nations Development Programme that classifies countries according to a social indicator comprising life expectancy, education, and income. The Human Development Index, in turn, is associated with the incidence and prevalence of certain types of cancer. The current state of knowledge shows that cancer varies greatly in its molecular characteristics and genetic expression. This has major implications for the early detection and treatment of the disease.

It is clear that prevention has proven effective, and today it is known that several types of cancer are preventable. Lifestyle changes (primary prevention) are an important tool in this regard, mainly the strengthening of tobacco control actions. However, the implementation of new forms or programs for early detection (secondary prevention) takes time to reach those in the earlier stages of cancer and to be translated into new therapeutic interventions to improve survival rates. Like other regions, Latin America needs to increase knowledge for the implementation and assessment of preventive and early detection policies. We must analyze how primary and secondary prevention can be accompanied by better timing of policies within the setting of health care services.

In terms of treatment, there are barriers that limit access for the region's most vulnerable groups who depend on public health care coverage.^3^ The barriers are a result of bureaucratic hurdles, lack of information, and suitable administration, which lead to underuse of available public resources. Many patients have erroneous conceptions regarding the treatments available as a result of disinformation or misrepresented information from different sources, or they lack the education that would empower them with respect to the health care system and their disease. There are patients who are forced to sell family assets or take out loans to be able to purchase the medicines they need, which has a serious impact on their personal wealth and even on their families' livelihoods. In addition, it is necessary to consider the limitations that patients who live in marginal regions with restricted access to health care systems must face. In these regions, higher mortality can result from insufficient access to the health care system for early diagnosis and specific treatments.

Regarding access to treatment, patient organizations play a key role by guiding the family in their search for resources available in public entities; informing patients of their rights and helping them assert these rights; making patients' demands visible and accompanying patients and families throughout the process; avoiding delays in administering adequate treatments while maintaining, whenever possible, a minimum reserve to respond to urgent needs; and by preparing guidelines to facilitate basic standardized information that is in an accessible language and is adapted to local culture and beliefs. There are issues related to the basic characteristics of cancer and the treatments available that are not included in the considerations affecting public health care coverage. For such issues, it is necessary to generate evidence from fieldwork to elevate the level of the discussion and improve the likelihood of success in controlling the disease.

With reference to the quality of care, there are no mechanisms or systems in Latin America that allow evaluating or measuring its impact. In any case, it is essential to create public policies that can assess the quality of oncologic care and that are adapted to the particulars of health care systems in each country. It is equally important to mobilize resources to develop, study, and assess these policies.

Finally, the care of survivors must be considered a guiding principle for controlling the disease. Survivors' reinsertion into the community and an active life and their role as an example for others should be encouraged through patient organizations. This can be done through testimonials to raise awareness and improve control of the disease as well as dispelling the myth of cancer as a death sentence. Increasing information by sharing people's experiences also has a positive impact on patient support and advocacy for the implementation of suitable public policies.

In May 2014, the Union for International Cancer Control and the Latin American and Caribbean Society of Medical Oncology jointly convened 19 medical experts and representatives of patient associations from nine Latin American countries in Bogota, Colombia, with a view toward promoting multisectorial cross-country knowledge sharing and collaboration among key actors in the area of oncology (Appendix). Discussions focused on the current state of cancer control in the region, main goals and challenges, and priority actions to improve prevention, diagnosis, access to treatment and care, and the quality of life of patients. Each topic on the agenda was analyzed from the perspectives of the individual and of health care institutions and a from a systemic viewpoint focused on public policies.

The Bogota meeting laid the foundations for the establishment of an independent, regional work group with many stakeholders, the Latin American Cancer Observatory, aimed at discussing and analyzing topics relevant to cancer in the region and offering recommendations to make progress against the disease. The conclusions of the first gathering drew attention to three key points for priority actions to improve cancer control in Latin America: first, reinforce aspects associated with screening and early detection of the disease and strengthening the education of health care professionals, patient groups, and health advocates in this area while developing processes oriented toward early diagnosis (at primary and intermediate levels of care). Second, improve access to treatment within the health care systems, ensuring that treatment occurs in the right way and in a timely manner. And third, promote holistic care and treatment by defining best practice standards, protocols, or guidelines for diagnosis and treatment that are adapted to Latin America, by fostering multidisciplinary support, and by introducing and supporting access to palliative care for patients with cancer as public policy.

The Observatory will continue with biannual meetings to follow the evolution of cancer control and define priorities for governments and civil society in the Latin American region.

## Appendix

*Members of the Latin American Cancer Observatory:* Argentina: Eduardo Cazap, Union for International Cancer Control and the Latin American and Caribbean Society of Medical Oncology; Ignacio Zervino, Fundación Atención Comunitaria Integral al Paciente Oncológico; Diego Paonessa, Liga Argentina de Lucha Contra el Cáncer; Gabriel Novick, Swiss Medical Group; Bolivia: Pablo Sitic Vargas, Instituto Oncológico del Oriente Boliviano; Brazil: Mauricio Magalhães Costa, Federación Latinoamericana de Mastología; Stephen Doral Stefani, Instituto do Cancer Mãe de Deus; Luciana Holtz, Instituto Oncoguia; Chile: Piga Fernández, Alianza GIST-Fundación Alianza de Tumores del Estroma Gastrointestinal; Jorge Madrid, Federación Latinoamericana de Sociedades de Cancerología; Colombia: Jairo Becerra, Fundación Alianza de Tumores del Estroma Gastrointestinal; Adriana Garzón, Fundación Simmon; Raúl Murillo Moreno, Instituto Nacional de Cancerología; Mexico: Mayra Galindo, Asociación Mexicana de Lucha Contra el Cáncer; Abelardo Meneses García, Instituto Nacional de Cancerología; Paraguay: José León Duarte, Instituto Nacional del Cáncer; Peru: Eva María Ruiz de Castilla Yabar, Esperantra; Venezuela: José Atias, Asociacion de Ayuda a Pacientes Hemato-Oncologicos; Yihad Khalek, Sociedad Venezolana de Oncología.
